# Snake-Eyes Appearance on MRI Occurs during the Late Stage of Hirayama Disease and Indicates Poor Prognosis

**DOI:** 10.1155/2019/9830243

**Published:** 2019-01-13

**Authors:** Haocheng Xu, Minghao Shao, Fan Zhang, Cong Nie, Hongli Wang, Wei Zhu, Xinlei Xia, Xiaosheng Ma, Feizhou Lu, Jianyuan Jiang

**Affiliations:** Department of Orthopedics, Huashan Hospital, Fudan University, Shanghai, China

## Abstract

**Purpose:**

Because Hirayama disease is stereotyped as a self-limited disease in the absence of a definite pathology, we investigated the potential relationship between snake-eyes appearance (SEA) and Hirayama disease to bring a new perspective in the pathological process of Hirayama disease based on relevant radiological and clinical evidence.

**Methods:**

A total of 30 cases observed SEA were selected from 293 patients with Hirayama disease to constitute the SEA group, and an equal number of cases were randomly selected from the remaining patients to form the non-SEA group. Cervical magnetic resonance imaging (MRI) was performed and subsequently used to measure the anteroposterior diameter and anterior shifting of the spinal cord. Additionally, clinical data, such as age, sex, duration of symptoms, symptoms, and signs, were collected and analyzed.

**Results:**

Of 293 patients, 10.6% appeared with the SEA, which was mainly multisegmental (86.7%), particularly at the C5-6 segment (73.3%), and intense with a well-defined border (70.0%). The SEA group was an older population (p < 0.0001) with a longer duration (p < 0.0001) and a higher incidence of Hoffmann signs and knee hyperreflexia (p < 0.0001, p = 0.0038, respectively). The degree of spinal cord atrophy demonstrated a close association with the SEA, as it was significantly worse in the SEA group and SEA segment (p = 0.0008, p < 0.0001, respectively). The degree of spinal cord atrophy was positively related to both age and duration (p = 0.0095, p = 0.0176, respectively).

**Conclusions:**

Confirmed as an irreversible lesion and an indication of poor prognosis, SEA appears during the late stage of Hirayama disease and is closely related to pyramidal signs and spinal cord atrophy.

## 1. Introduction

Hirayama disease is an insidious distal upper limb weakness and atrophy, which occurs mainly in young males [1]. Classical magnetic resonance imaging (MRI) findings of Hirayama disease include the following: (1) an anterior shifting of the spinal cord with forward compression and focal atrophy in the lower cervical cord; and (2) anterior shifting of the posterior dura and the resulting venous plexus dilatation appearing as a crescent or focal mass with flow voids in the enlarged posterior epidural space displaying strong contrast enhancement [2, 3].

**Figure 1:**
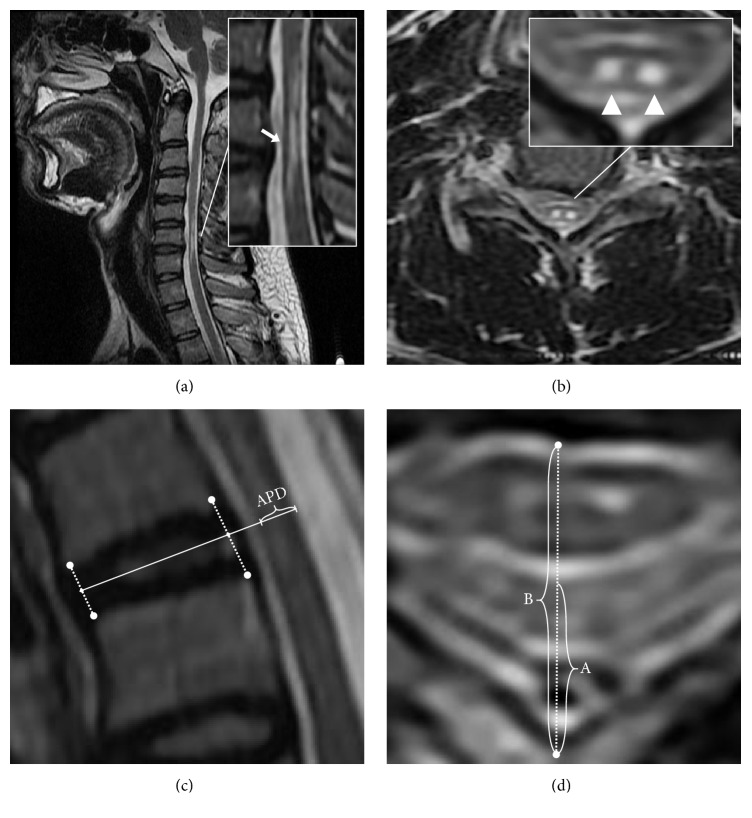
Typical SEA and measurement method on MRI. (a) On the sagittal T2-weighted MRI, the SEA showed a high signal in the middle of the spinal cord. (b) On the axial image, a typical SEA was characterized as a symmetrical, bilateral, small high-signal-intensity lesion. (c) Measurement of anteroposterior diameter (APD) on the sagittal image: APD is the distance between the anterior and the posterior edge of the spinal cord on the mid-disc line. (d) Measurement of spinal cord anterior shifting on the axial image: the distance between the posterior wall of the spinal canal and the posterior/anterior edge of the cervical spinal cord was termed A/B. Then, the ratio of A to B was used as an indicator of anterior shifting.

Snake-eyes appearance (SEA) is a unique radiological finding characterized as a symmetrical bilateral small high-signal-intensity lesion on an axial T2-weighted MRI and is named because of its similar appearance to the face of a snake (Figures 1(a) and 1(b)). Some authors suggested that SEA is a reversible change, similar to edema or gliosis [4–6]. Instead, its pathologic result is cystic necrosis at the junction of the central gray matter near the ventrolateral posterior column [7].

The presence of SEA in Hirayama disease is not uncommon. Furthermore, we found that Hoffmann signs and knee hyperreflexia could appear in these patients, particularly with the SEA on MRI, which is contrary to the conventional view that Hirayama disease is a self-limited disease in the absence of pyramidal signs [8]. Therefore, there is an urgent necessity to better understand the relationship between Hirayama disease and SEA and to reconsider the pathological process of Hirayama disease based on relevant radiological and clinical evidence.

## 2. Materials and Methods

### 2.1. Patient Population

Between January 2009 and July 2017, 293 patients met the diagnostic criteria of Hirayama disease and were treated in the Spinal Surgery Center of Huashan Hospital Affiliated with Fudan University. After review of the 293 patients, we enrolled all 30 cases who revealed SEA on the axial T2-weighted MRI as the SEA group. We selected an equal number of cases from the remaining patients as the non-SEA group using the random sampling method.

The diagnostic criteria for Hirayama disease [9] were as follows: (1) insidious disease onset, (2) unilateral or asymmetric weakness and amyotrophy of the distal upper extremities without sensory dysfunction or lower limb involvement with denervation that was limited to the unilateral or bilateral upper extremities and was identified using electrophysiological methods in addition to normal sensory nerve function, (3) with or without clinical symptoms, such as “cold paralysis”, “fasciculation”, and “tremor in the upper extremities”, and (4) flexion cervical MRI showing lower cervical compression that resulted from forward shifting of the posterior dura and a crescent abnormality posterior to the dura.

### 2.2. Clinical Characteristics

Reviewing the medical histories of the 60 patients, we collected relevant clinical data, such as age, sex, duration of symptoms, symptoms, and signs. The age was recorded by years and the durations by months. For the symptoms and signs, the side of weakness and amyotrophy, Hoffmann sign and knee-jerk reflex were evaluated.

### 2.3. Imaging Parameters

To diagnose Hirayama disease, all patients should have a neutral position and flexion position cervical MRIs. Flexion position MRI was performed following the protocol suggested by Hirayama and Tokumaru [10]. With the support of a wedge pillow, patients will achieve a full neck flexion by drawing the chin close to the chest. The degree of neck flexion is about 45 degrees but may have minor adjustments depending on the patient's body size and tolerance. All MRIs were performed using a GE Signa HDxt 1.5T MR Scanner (GE Healthcare, Milwaukee, WI, USA). The center of the magnetic field was set at the C5 level. The routine spin-echo sequence was used for scanning to obtain sagittal T1-weighted and T2-weighted images and axial T2-weighted images. For the axial T2-weighted images, the scanning plane line was parallel to the intervertebral space. The parameters of sagittal T1-weighted images were FOV = 24*∗*24cm, TR/TE = 620ms/13ms, thickness = 3.0mm, and matrix = 288*∗*160. The parameters of sagittal T2-weighted images were FOV = 24*∗*24cm, TR/TE = 2680ms/128ms, thickness=3.0mm, and matrix = 320*∗*160. The parameters of axial T2-weighted images were FOV = 20*∗*20cm, TR/TE = 2000ms/99ms, thickness = 4.0mm, and matrix = 288*∗*160. The scanning image was stored in the PACS system (Centricity 3.0, General Electric Medical System, Milwaukee, Wisconsin, USA). All the parameters were measured three times, and the average was used for each data point.

In the SEA group, the location and classification of the SEA were recorded on the central sagittal T2-weighted MRI based on the principles reviewed by Vedantam et al. [11] (Table 1). In the multisegment SEA cases, the segment of the SEA was recorded as the most obvious segment of increased signal intensity.

The imaging parameters of the corresponding C3-4, C4-5, C5-6, C6-7, and C7-T1 segments were measured. On the sagittal image, a straight line is formed connecting the midpoint of the anterior edge of the intervertebral disk with the midpoint of the posterior edge of the intervertebral disk. The distance between the anterior and posterior edge of the spinal cord on this straight line was measured and used as the anteroposterior diameter (APD) of the spinal cord (Figure 1(c)). To reduce individual differences, the APD of the spinal cord at the C2-3 level was measured as an internal reference standard because the spinal cord at this level was usually free from compression. Then, the APD of the target segments was divided by that of C2-3, and the relative value was used as the parameter representing spinal cord atrophy. The most severe segment of spinal cord atrophy was selected as representative for the intergroup comparison.

On the axial image, the distance between the posterior edge of the spinal cord and posterior wall of the spinal canal was termed A, and the distance between the anterior wall and posterior wall of the spinal canal was termed B. Then, the ratio of A to B was used as an indicator for the anterior shifting of the spinal cord (Figure 1(d)) [12]. The most severe segment of anterior shifting was selected as representative for the intergroup comparison.

### 2.4. Statistical Analyses

Statistical analyses were performed using the IBM SPSS version 20.0 (SPSS, Chicago, IL, USA). Statistical analyses of sex, Hoffmann sign, and knee-jerk reflex were performed using Fisher's exact test, whereas the analysis of the atrophy side was performed using the chi-square test. The independent sample t-test was used for statistical analyses of the age, duration, spinal cord atrophy, and anterior shifting between the two groups. A multivariable analysis was performed to determine the factors that were independently associated with the SEA. Statistical analyses of the spinal cord atrophy and anterior shifting among the different segments in the SEA group were performed using a one-way analysis of variance. The relationship for age, duration, and imaging parameters were analyzed using a linear regression. P < 0.05 was defined as statistically significant.

## 3. Results

### 3.1. Patient Population

A total of 60 subjects were included (58 males, mean age 21.55 years, range 15-44 years). Of the 293 cases treated in our department, 31 cases revealed SEA. The incidence of the SEA in HD is 10.6%. One SEA patient was excluded because his radiographic data could not be measured.

### 3.2. Characteristic of SEA

In the SEA group, SEA mainly occurred at the C5-6 segment in a total of 22 (73.3%) patients. The C4-5 segment accounted for 6 patients (20.0%), and the C6-7 segment accounted for 2 patients (6.7%). According to the intramedullary increased signal intensity classification method shown in Table 1, 26 (86.7%) cases presented multisegment SEA (2 segments in 19 cases, 3 segments in 5 cases, 4 segments in 1 case, and 5 segments in 1 case), whereas SEA was limited at a single segment level in 4 (13.3%) cases. In addition, the SEA signal strength was intense with a well-defined border in 21 (70.0%) cases, and the SEA was faint with a fuzzy border in 9 (30.0%) cases.

### 3.3. Clinical Findings

The demographic and clinical characteristics of the 60 patients are presented in Table 2. The average age of the SEA group was (24.40 ± 1.21) years and was significantly higher than that of the non-SEA group (18.70 ± 0.46, p < 0.0001, Figure 3(b)). The mean duration of disease of the SEA group was (72.80 ± 10.93) months and significantly longer than that of the non-SEA group (21.07 ± 3.31, p < 0.0001, Figure 3(c)). There was no significant difference in sex or atrophy side distribution between the two groups (p = 0.4915 and p = 0.4172, respectively). In the physical examination, the SEA group presented a higher incidence of pyramidal signs than that in the non-SEA group, such as Hoffmann signs and knee hyperreflexia (p < 0.0001 and p = 0.0038, respectively). By multivariable analysis, the duration of disease and positive Hoffmann sign were independent predictors of the occurrence of the SEA.

### 3.4. Radiological Finding

**Figure 2:**
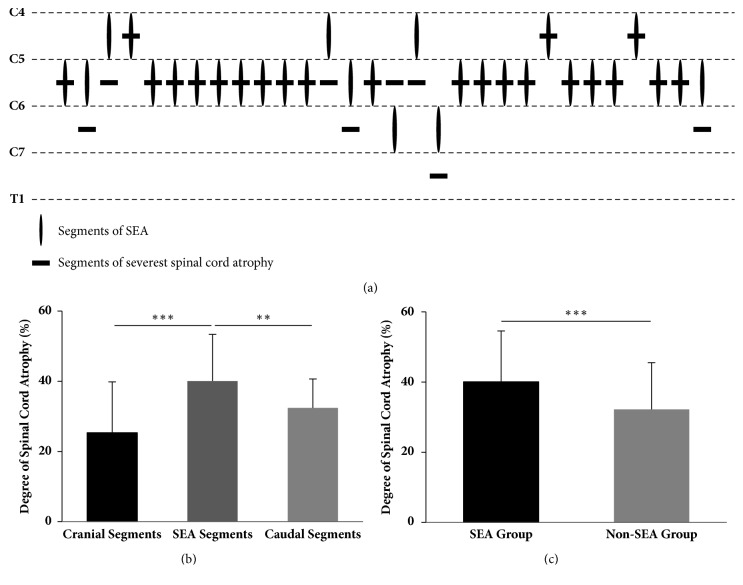
The association between the degree of spinal cord atrophy and SEA. (a) The most intense SEA signal occurred at the C5-6 segment in 22 (73.3%) patients, C4-5 in 6 (20.0%) patients, and C6-7 in 2 (6.7%) patients. The segment of the SEA was matched with that of the severest spinal cord atrophy in 22 (73.3%) cases. (b) In the SEA group, the spinal cord atrophy of SEA segments was significantly worse than that in the cranial adjacent segments and caudal adjacent segments (*∗∗∗* denotes p < 0.001, *∗∗* denotes p < 0.01, one-way analysis of variance). (c) Between two groups, the severity of spinal cord atrophy in the SEA group was significantly higher than that in the non-SEA group (*∗∗∗* denotes p < 0.001, t-test).

The segment of the SEA was matched with that of the severest spinal cord atrophy in 22 (73.3%) cases (Figure 2(a)). Additionally, the spinal cord atrophy of SEA segments was significantly worse than that of cranial adjacent segments and caudal adjacent segments (p < 0.0001, Figure 2(b)). Moreover, the severity of spinal cord atrophy in the SEA group was significantly higher than that in the non-SEA group (p = 0.0008, Figure 2(c)), and it was also demonstrated as an independent predictor of the SEA by the multivariable analysis. However, there was no significant difference in the degree of spinal cord anterior shifting between the two groups (p = 0.0792, Table 3).

### 3.5. Relationships between Age, Duration, and Imaging Appearances

**Figure 3:**
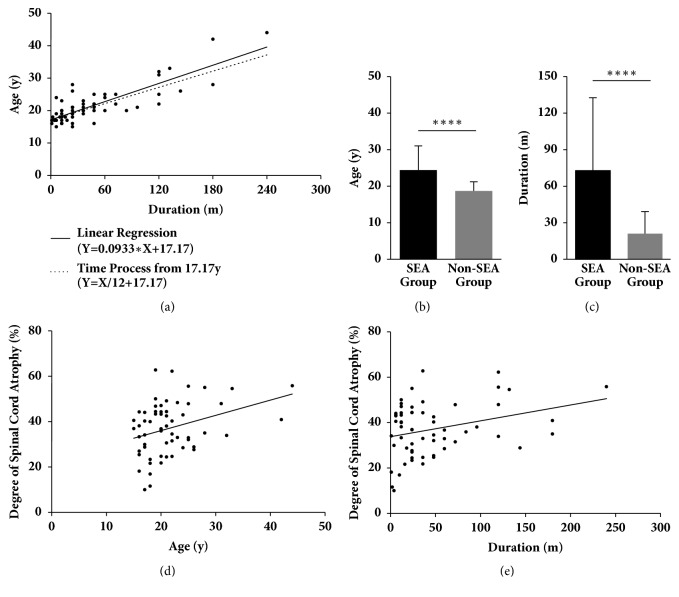
The deterioration of spinal cord atrophy and SEA were time-dependent. (a) The duration of disease was positively related to the ages of patients (F = 126.4, p < 0.0001, linear regression). The line of the linear regression (Y=0.0933*∗*X+17.17) closely resembles the line of the natural aging process from 17.17 years (Y=1/12*∗*X+17.17). (b) and (c) Both the age and duration in the SEA group were significantly higher than that in the non-SEA group (p < 0.0001 and p < 0.0001, respectively, t-test). (d) and (e) The degrees of spinal cord atrophy demonstrated positive correlations with the patients' ages (p = 0.0095, F = 7.207, linear regression) and duration of disease (p = 0.0176, F = 5.971, linear regression); therefore, spinal cord atrophy progressed gradually with time.

The duration of disease was positively related to the age of patients (p < 0.0001, Figure 3(a)) and the line of the linear regression closely resembled the natural time process from the age of 17.17 years.

The deterioration of spinal cord atrophy was time-dependent, as the degree of spinal cord atrophy demonstrated positive correlations with the patient's age (p = 0.0095, Figure 3(d)) and duration of disease (p = 0.0176, Figure 3(e)). Additionally, there were no significant correlations between the degree of spinal cord anterior shifting and either age (p = 0.7281) or duration of disease (p = 0.0765).

## 4. Discussion

The description of the SEA was initially given in a CT-myelography study of 7 cervical spondylotic myelopathy patients in 1980s [13]. Mizuno et al. [7] then confirmed the pathology results of the SEA by autopsy and found that cystic necrosis at the junction of the central gray matter and the ventrolateral posterior column and loss of anterior horn cells were the main changes. However, the definite pathological process of the SEA in chronic spinal cord compression remains ambiguous. Potential mechanisms, such as chronic mechanical compression, secondary changes in blood supply, and the high vulnerability of gray matter cells, may contribute to the process [14]. With advances in MRI techniques, increasing numbers of patients with chronic compressive myelopathy, such as cervical spondylotic myelopathy and ossification of the posterior longitudinal ligament, are observed with the SEA on MRI reports. In our clinical practice, we discovered that SEA occurred in 10.6% of Hirayama disease patients.

Hirayama disease is stereotyped as a self-limited disease without pyramidal signs such as Hoffmann's signs and knee hyperreflexia [1]. But in our results, pyramidal signs could be observed in these patients, particularly with SEA showing on the MRI, which may indicate the occurrence of spinal cord injury or cervical spondylotic myelopathy. Thus we hypothesized that Hirayama disease has the possibility of progressing to cervical spondylosis. Accompanied by several serious symptoms, cervical spondylotic myelopathy is not a self-limiting disease like Hirayama disease. So we believe that the appearance of pyramidal signs indicates the deterioration of the disease and also prompts the poor prognosis. Due to the close relation to the appearance of pyramidal signs, SEA is believed to be one of the MRI findings indicating poor prognosis in Hirayama disease.

As intramedullary high signals were more inclined to appear in severe compression cases with cervical spondylotic myelopathy [15], we hypothesize whether the emergence of the SEA is related to the degree of anterior compression, which plays a very important role in the pathogenesis of Hirayama disease. The result showed that SEA mainly appears at the C5-6 segment, surprisingly consistent with the segment prone to anterior compression in Hirayama diseases. In up to 73.3% of patients, the segment of the SEA was matched with that of the severest anterior compression. Moreover, the spinal cord atrophy of SEA segments was significantly worse than that of the cranial and caudal adjacent segments, and the SEA group showed more serious spinal cord atrophy than did the non-SEA group. This result indicates that there is a clear correlation between SEA and spinal cord atrophy. Spinal cord atrophy means the possibility of spinal cord ischemia and spinal cord injury, so this finding also proved that SEA is one of the hints that the patient's prognosis is poor.

Because the initiating factor of Hirayama disease is a dynamic mechanism associated with a marked narrowing of the anterior subarachnoid space, widening of the posterior, and even direct contact of the spinal cord with the anterior spinal wall upon flexion, we collected data on the extent of spinal cord anterior shifting in both the SEA and non-SEA groups. However, there was no significant difference between the two groups. A previous study also suggested that the increased signal intensity may be irrelevant with dural shifting [16]. Therefore, changes in spinal cord dynamics in Hirayama disease may be more complicated than expected. We questioned the mechanism underlying the divergent results that occurred while a similar extent appeared on anterior shifting; therefore, we focused on the effect of time.

As the imbalanced development of the spinal cord and dura plays an essential role in pathogenesis [17], the onset age of Hirayama disease generally peaks in adolescence, which is a period of rapid growth. We also confirmed this because the duration of disease was positively related to age. Furthermore, the line of the linear regression closely resembled the natural time process from the age of 17.17 years, indicating that the onset age of Hirayama disease is approximately 17 years old. Thus, the difference in both the age and duration of disease between the SEA and the non-SEA group may profile the duration of compression. As a result, the duration of compression in the SEA group was markedly longer than that in the non-SEA group. In addition, both the age and duration were significantly linear relative to the atrophy of the spinal cord. Our results indicated that the atrophy was aggravated with the progression of the disease. SEA and pyramidal signs may form by degrees in some cases, and these patients may develop a poor prognosis.

The changes in the dural sac and spinal cord during the pathological process of Hirayama disease have not been explicitly clarified. Here, we base our hypothesis on our latest clinical data. Because of a disproportional elongation of the vertebral canal, the posterior dural sac is comparatively shorter, resulting in a tightened and anteriorly displaced dural canal during flexion, which then induces an anterior compression of the spinal cord against the anterior wall of the spinal canal [15, 18]. Repeated or sustained compression brings the anterior spinal cord into chronic circulatory insufficiency, which was supported by autopsy and radiological studies [8, 19]. Because it is vulnerable to ischemia and hypoxia, the central gray matter of the ventrolateral posterior column becomes the most frequent part impaired [20]. Presumably, vascular insufficiency secondary to the long-term mechanical compression may cause the formation of focal necrotic lesions and cavities after phagocytosis in the gray matter, appearing as the SEA in imaging [21]. To further verify the morphology of SEA, we also found pencil-shaped SEA in most of our Hirayama patients, which was demonstrated by Al-Mefty as a longitudinal extending of the cystic necrosis lesion in the central gray matter and resulted from repeated or sustained compression [22].

For the treatment of Hirayama disease, we propose several treatment concepts based on our new findings. Firstly, SEA is an irreversible destruction of the gray matter accompanied with significant neuronal loss in the anterior horn [7] and also is an unfavorable prognostic factor for the recovery of upper-extremity motor weakness [7]. It is not common in Hirayama disease, but as the prolongation of the disease course, the occurrence rate of the SEA is getting increasingly high, which suggests that the disease has developed in the direction of irreversible spinal cord injury. This is different from the traditional understanding of Hirayama disease, so the emergence of SEA deserves our attention. Secondly, the imaging characteristics of the SEA in Hirayama disease patients were multisegmental and ‘sharp, intense'. As reported by Vedantam et al. [11], these kinds of high signal intensity are associated with poorer functional outcome than other types of T2-weighted increased signal intensity on MRI images. It suggests that we need to make the intervention in advance to avoid the emergence of the SEA. Finally, Hirayama disease is considered as a self-limited disease due to less arterial compression after the progression of local cord atrophy and less or no dural shifting in long course patients [2], and it often stops progressing after 5 years of onset. We acknowledge that Hirayama disease may gradually be stable in the imaging changing and symptoms of unilateral muscular atrophy. However, we observed an average duration of 72 months in the SEA group, which means that the SEA may be observed in these patients with the company of pyramidal signs after 6 years of onset. Considering these evidence, we cannot be sure that Hirayama disease is self-limiting, and it even has the possibility of developing in the direction of cervical spondylosis. Thus, it is recommended that surgical intervention is performed in a timely matter for patients with Hirayama disease, particularly with SEA sign, considering either the recovery of motor function or blocking of myelopathy progression.

## 5. Limitations

This is a retrospective observational case-control study, and we cannot conclude that the SEA is correlated with an unfavorable prognosis. Further prospective, large sample clinical studies are necessary to confirm the relationship between the SEA and prognosis. Another limitation is the position of the MRI examination. The position of the cervical cord and the degree of cord compression upon flexion may also vary depending on different positions. Although an orthosis was used, the degree of neck flexion was not completely consistent. More significant anterior cord compression could be seen when patients were placed in a hyperflexion position because of gravity.

## 6. Conclusions

SEA appeared in the late disease of Hirayama disease, and it was closely related to pyramidal signs and spinal cord atrophy. Confirmed as an irreversible lesion, SEA signifies a poor prognosis and could be the result of chronic compression and secondary vascular insufficiency as we hypothesized. Hirayama disease shows pyramidal signs and a tendency to be non-self-limiting when SEA appears, which is different from conventional concepts. Therefore, timely surgical intervention is necessary.

## Figures and Tables

**Table 1:** Types of classifications of T2-weighted increased signal intensity on MR images.

**Type of classification (Basis of classification)**	**Grade**	**Description**
Longitudinal extent of ISI (One or more segments)	0	No ISI
	1	Focal/single segment ISI
	2	Multisegmental ISI
Qualitative (ISI intensity and border)	0	No change
	1	Faint, fuzzy border
	2	Intense, well-defined border

ISI: Increased signal intensity		

**Table 2:** Demographics and imaging parameters of 60 patients with HD.

**Characteristic**	**SEA**	**Non-SEA**	**Univariate analysis**	**Multivariate analysis**		
				**OR**	**95**%** CI**	**p-value**
Age (y)	24.40 ± 1.21	18.70 ± 0.46	**p < 0.0001 (t = 4.395)**			
Sex (no. [%])						
Male	28 (93.3)	30 (100)				
Female	2 (6.7)	0 (0)	p = 0.4915			
Duration (m)	72.80 ± 10.93	21.07 ± 3.31	**p < 0.0001 (t = 4.532)**	0.960	0.928-0.993	**0.016**
Atrophy side (no. [%])						
Left	10	8				
Right	11	16				
Bilateral	9	6	P = 0.4172 (*χ*^2^ = 1.748)			
Hoffmann sign (no. [%])						
(-)	14 (46.7)	28 (93.3)		1 (ref)		
(+)	16 (53.3)	2 (6.7)	**p < 0.0001**	7.200	1.197-43.310	**0.031**
Knee-jerk reflex (no. [%])						
(++)	11 (36.7)	23 (76.7)				
(+++)/(++++)	19 (63.3)	7 (23.3)	**p = 0.0038**			
Atrophy (%)	41.93 ± 1.95	32.22 ± 1.93	**p = 0.0008 (t = 3.534)**	0.917	0.843-0.998	**0.044**
Shifting (%)	52.76 ± 1.82	56.61 ± 1.15	p = 0.0792 (t = 1.786)			

**Table 3:** Relative anteroposterior diameters and anterior shifting of the spinal cord in 60 patients with HD.

**Parameter**	**SEA**	**Non-SEA**	
Relative anteroposterior diameters	0.592 ± 0.021	0.658 ± 0.021	**t=2.188 (p = 0.0327)**
Anterior shifting	0.472 ± 0.018	0.434 ± 0.011	t=1.790 (p = 0.0787)

## Data Availability

The data used to support the findings of this study are available from the corresponding author upon request.
